# Preference for Well-Balanced Saliency in Details Cropped from Photographs

**DOI:** 10.3389/fnhum.2015.00704

**Published:** 2016-01-11

**Authors:** Jonas Abeln, Leonie Fresz, Seyed Ali Amirshahi, I. Chris McManus, Michael Koch, Helene Kreysa, Christoph Redies

**Affiliations:** ^1^Experimental Aesthetics Group, Institute of Anatomy I, University of Jena School of Medicine, Jena University HospitalJena, Germany; ^2^Computer Vision Group, Institute of Computer Science, Friedrich Schiller UniversityJena, Germany; ^3^Division of Psychology and Language Sciences, University College LondonLondon, UK; ^4^General Psychology and Cognitive Neuroscience, Institute of Psychology, Friedrich Schiller UniversityJena, Germany

**Keywords:** experimental aesthetics, photography, visual saliency, eye movements, visual balance, statistical image properties, bottom-up processing

## Abstract

Photographic cropping is the act of selecting part of a photograph to enhance its aesthetic appearance or visual impact. It is common practice with both professional (expert) and amateur (non-expert) photographers. In a psychometric study, McManus et al. (2011b) showed that participants cropped photographs confidently and reliably. Experts tended to select details from a wider range of positions than non-experts, but other croppers did not generally prefer details that were selected by experts. It remained unclear, however, on what grounds participants selected particular details from a photograph while avoiding other details. One of the factors contributing to cropping decision may be visual saliency. Indeed, various saliency-based computer algorithms are available for the automatic cropping of photographs. However, careful experimental studies on the relation between saliency and cropping are lacking to date. In the present study, we re-analyzed the data from the studies by McManus et al. (2011a,b), focusing on statistical image properties. We calculated saliency-based measures for details selected and details avoided during cropping. As expected, we found that selected details contain regions of higher saliency than avoided details on average. Moreover, the saliency center-of-mass was closer to the geometrical center in selected details than in avoided details. Results were confirmed in an eye tracking study with the same dataset of images. Interestingly, the observed regularities in cropping behavior were less pronounced for experts than for non-experts. In summary, our results suggest that, during cropping, participants tend to select salient regions and place them in an image composition that is well-balanced with respect to the distribution of saliency. Our study contributes to the knowledge of perceptual bottom-up features that are germane to aesthetic decisions in photography and their variability in non-experts and experts.

## Introduction

Since the invention of photography by Nicéphore Niépce and Louis Daguerre in the early 19th century, a central issue in photography has been how to choose a suitable viewpoint and a proper viewing window, through which a small part of the real world is captured on a photograph in a visually pleasing way. With the recent rise of affordable digital cameras, this question has become of interest not only to professional photographers but also to a wide audience of laypersons who take photographs casually on many occasions.

A task closely related to choosing a good photographic detail is the act of selecting part of a photograph that already exists (photographic “cropping”), to enhance its visual impact or its aesthetics. Cropping is not only common practice for professional photographers, but also non-expert photographers crop their photographs at home with the help of widely available computer programs, such as Adobe Photoshop. In a conceptual sense, the taking of the original photograph can be seen as “cropping the visual world” through the viewfinder or screen of the camera.

In a first experimental study on the psychometrics of photographic cropping, McManus et al. (2011b) studied how reliably people crop photographs, how much variability there is between individual croppers in the quality of cropping decisions, and whether expert (professional) photographers crop differently from non-experts. In their study, participants cropped every-day photographs, which were displayed on a computer screen in a laboratory setting, to half of their linear size (1/4 of the area). The study revealed that both experts and non-experts cropped photographs confidently and reliably. Additionally, independent observers preferred aesthetically the crops of some participants over those of other croppers, suggesting individual differences in expertise. Experts tended to select details from a wider range of possible cropping positions than non-experts, but judges did not generally prefer the expert crops.

Although subjectively, most people feel rather confident about how to crop a photograph (McManus et al., 2011b), the criteria, on which experts and non-experts ground their cropping decisions, are not well understood. Without any doubt, one major issue for cropping a photograph is which key objects or parts of a scene people regard as essential to be included in the photograph. Besides such content-based cropping criteria, some professional photographers and psychologists claim that other criteria, which relate to formal rules of image composition, should also be followed (Arnheim, 1954, 1982; Palmer et al., 2008; Liu et al., 2010). In photography, several compositional rules were made explicit (Hicks, 2005) and some of these rules have been scrutinized recently at the experimental level.

For example, Bruno et al. (2014) studied three well-known principles of photographic composition (the rule of thirds, the golden ratio rule and the eye centering principle) in self-portraits taken with a hand-held smartphone camera (“selfies”). The rule of thirds postulates that images are aesthetically more pleasing if important compositional elements are placed close to one of the third lines of the image. The golden ratio rule says that rectangular images are preferred aesthetically if the ratio of the larger side over the smaller side (a/b) equals the ratio of the sum of the two sides over the larger side (a/[a+b]), i.e., if the ratio is about 1.618. The eye centering principle claims that, in portrait images, one of the eyes of a depicted person should be centered horizontally. Bruno et al. (2014) found that non-professional photographers do not follow any of these rules. In another recent study, Amirshahi et al. (2014) investigated the rule of thirds in a large set of photographs and paintings and did not detect any preference for images that followed the rule of thirds, compared to those that did not.

Another principle commonly used to assess photographic quality is visual saliency (Frintrop et al., 2010). The term saliency denotes any number of properties that make an object or feature stand out from its background; such properties can therefore attract visual attention and direct the observer’s gaze to a particular (salient) region of an image. The properties that confer saliency to image regions can be tied to cognitive factors, emotional value, or goals (Henderson et al., 2007; Frintrop et al., 2010; Niu et al., 2012), but they can also be visual characteristics, such as color, luminance, contrast, and spatial frequency. In the present work, we use computer-based algorithms that are based on low-level visual characteristics to measure visual salience and to determine how well such characteristics determine cropping behavior. There are many different algorithms for calculating visual saliency and each of them uses a different set of low-level visual features to predict eye movements. In an exhaustive survey of these algorithms, Borji et al. (2013) found that the performance of 35 state-of-the-art models differed between each other and depended on the respective task. Because the magnitude of the bottom-up contribution to active gaze control is still controversial, we assess the performance of two saliency models by comparing computed saliency maps with experimentally measured eye movements in the present study.

Finally, the century-old concept of pictorial balance plays a role in the subjective evaluation of visual stimuli, including photographs and artworks (for example, see Ross, 1907; Howard, 1914; Arnheim, 1954). Pictorial balance is thought to unify picture elements into a cohesive composition. In a recent overview on pictorial balance, Gershoni and Hochstein (2011; p. 509) stated that “balance is achieved by structural properties working like mechanical weights with a fulcrum at the picture’s center, on which an imaginary lever is poised, so that heavy weights can be counterbalanced by lighter ones located further from the center”. In the Arnheim-Ross model of visual balance, the framework of these levers is set on the major geometrical axes (vertical, horizontal and diagonal) that intersect in the image center (Ross, 1907; Arnheim, 1954). For example, the image is thought to be balanced on the horizontal axis if as much force is present on the right side as on the left side of the axis (image) center.

Relatively few studies have linked the subjective impression of pictorial balance to objective physical properties. In a follow-up to their cropping study, McManus et al. (2011a) did not find any evidence for a correlation of cropping selections with the Arnheim-Ross model of visual balance. As an objective measure of balance for a given physical property, such as luminance, the center-of-mass has been used. It represents the unique point where the weighted distributed mass of the property is equal on either side of an image axis; an image is more balanced if this point is located closer to the geometrical image center. Jahanian et al. (2015) studied pictorial balance by modeling visual weight with the saliency of low-level visual features. For a large set of aesthetically pleasing photographs, they obtained results compatible with Arnheim (1954) concept of major axes of composition, including the relevance of the image center. Besides the structural properties mentioned above, there may be additional formal rules that are followed intuitively and await a description in physical or perceptual terms.

Despite the overall difficulties in explaining the quality of photographic composition by unique and simple rules, there is evidence that combinations of multiple low-level image properties can be used to predict the aesthetic outcome of photographic cropping. This evidence stems mostly from the field of computational aesthetics, where researchers used large datasets of photographs posted on websites, such as *Photo.net* or *Flickr.com*, which have been rated by the photographic community. For example, Tong et al. (2004) were among the first to successfully use a set of low-level image features in an image classification task that distinguished between professional photographs and photographs by home users. Their classifier also predicted the quality ratings by human observers with relatively high accuracy. Datta et al. (2006) trained an automated classifier to distinguish between high- and low-rating photographs, based on 56 low-level image features that related to rules of thumb and common intuition in photography, such as colorfulness, the rule of thirds, various texture and shape measures, size and aspect ratio, and depth of field. With a short-list of 15 of these measures, they achieved an accuracy rate of around 70% in predicting highest/lowest ratings of a large dataset of 1664 photographs. Wong and Low (2009) used a saliency-enhanced approach for the classification of professional photographs and snap-shots. With this higher-level approach, they achieved classification rates of up to 79%. Sun et al. (2009) integrated top-down supervision and personalized parameters into a bottom-up attentional model to predict image quality. Low-level statistical image properties have also been studied in artworks (for reviews, see Graham and Redies, 2010; Redies, 2015).

Based on saliency calculations of low-level image properties, a large number of computational tools have been devised to carry out cropping decisions automatically (also called *image retargeting*; for reviews, see Vaquero et al., 2010; Ardizzone et al., 2013). For example, Ardizzone et al. (2013) compared five different saliency algorithms for automatic cropping and obtained favorable results with all of them. Two other studies on saliency-based cropping applications were published by Suh et al. (2003) who shrank original images to produce easily recognized thumbnails for image retrieval, and by Ciocca et al. (2007) who redesigned large images for small screens with an adaptive visual attention model that incorporated semantic information. Santella et al. (2006) introduced an interactive method by which saliency-based cropping was combined with information about important image content that was obtained from eye tracking data. They demonstrated experimentally that viewers prefer gaze-based crops to fully automated crops. Liu et al. (2010) combined several compositional rules to derive an aesthetic measure for evaluating cropped photographs. Other studies that have used similar approaches are too numerous to be discussed here in detail.

Although saliency-based cropping methods are used widely, relatively few psychological studies have investigated the role of saliency in cropping decisions. In the present work, we re-analyzed the experimental data that were obtained by McManus et al. (2011a,b). To assess the relevance of the saliency maps for gaze control, we obtained maps of eye fixations for comparison.

In the studies by McManus et al. (2011a,b), participants selected a large number of rectangular details from a series of every-day photographs (see above). In addition to the two sets of details that were selected by non-experts and experts (here called *selected* details), we systematically examined all details that were not selected during the cropping of the same photographs (here called *avoided* details). We asked the following questions:

1. Did participants select details during cropping that contained higher overall saliency and attracted longer eye fixations, compared to the avoided details? We anticipated that this would be the case because participants are more likely to select image regions that attract their attention during the cropping procedure (Ardizzone et al., 2013).
2. Did participants select details that had a higher pictorial balance of saliency and of total dwelling (fixation) time than avoided details (as assumed, for example, by Liu et al., 2010; Chen et al., 2013; Wang et al., 2015)? As a measure of pictorial balance, we determined how close the center-of-mass values for visual saliency and for total dwelling times were to the geometrical image center of the details.
3. Do regularities in cropping decisions depend on the recognition of image content? To answer this question, we analyzed versions of the original photographs that had been transformed to binarized, monochrome images, in which the brighter parts of the image were rendered white and the darker parts black (Mooney, 1956; here called *Mooney images*; for details of their generation, see McManus et al., 2011b). This manipulation made it very difficult to recognize image content. McManus et al. (2011b) reported that the Mooney versions of the photographs showed dramatically altered cropping positions, but were still cropped consistently. The authors suggested that image structure, perhaps in the form of low-level image properties may explain this consistency (McManus et al., 2011b). We therefore asked whether cropping decisions for Mooney images were based on a similar pattern of low-level image properties and eye movements as the cropping of the original color photographs.
4. Did the selected details differ between non-expert participants and expert (professional) participants in any of these measures? We expected to find differences because expert photographers were previously found to crop photographs differently from non-experts (McManus et al., 2011b).

To compute visual saliency in the present study, we used two algorithms, the Itti-Koch model (Itti et al., 1998) and the graph-based visual saliency model (Harel et al., 2007; for details, see “Materials and Methods” Section). The two methods will be referred to as ITTI and GBVS, respectively, in the remainder of this work. Moreover, selected and avoided details were carefully matched to avoid possible artifacts due to a central tendency, which is inherent in both eye gaze patterns and saliency calculations (Harel et al., 2007; Tatler, 2007; Foulsham and Underwood, 2008; Bindemann, 2010). There is a central tendency in gaze patterns because observers tend to fixate the central regions of an image more than its peripheral regions, irrespective of the distribution of image content. Likewise, in the saliency maps calculated with ITTI and GBVS, saliency falls off towards the edges of the image. Both central tendencies were controlled for by analysing pairs of selected and avoided details with matched distances between their centers and the geometrical image center of the original photographs.

## Materials and Methods

### Datasets of Images

The present work re-analyzed a series of four datasets of cropped photographs; some but not all of the data were reported in the studies by McManus et al. (2011a,b). Specifically, the first dataset corresponded to the 20 croppers described in Study 1 of McManus et al. (2011b). The second dataset was obtained from the 41 non-expert and 10 expert participants described in Studies 3, 4 and 5 of McManus et al. (2011b), with the cropping data corresponding to Study 4 of that publication. The third dataset consisted of 36 participants who took part in Study 5 of McManus et al. (2011a), although not all of their data were reported there. Finally, a fourth dataset was from of 38 participants, who took part both in Study 6 of McManus et al. (2011b), and study 3 of McManus et al. (2011a), their cropping data being analyzed only in the latter. The four groups carried out 80 separate croppings, although not always of the same images, as different hypotheses were being investigated. All subjects carried out some repeat croppings of some images to assess reliability (see below).

**Figure 1:**
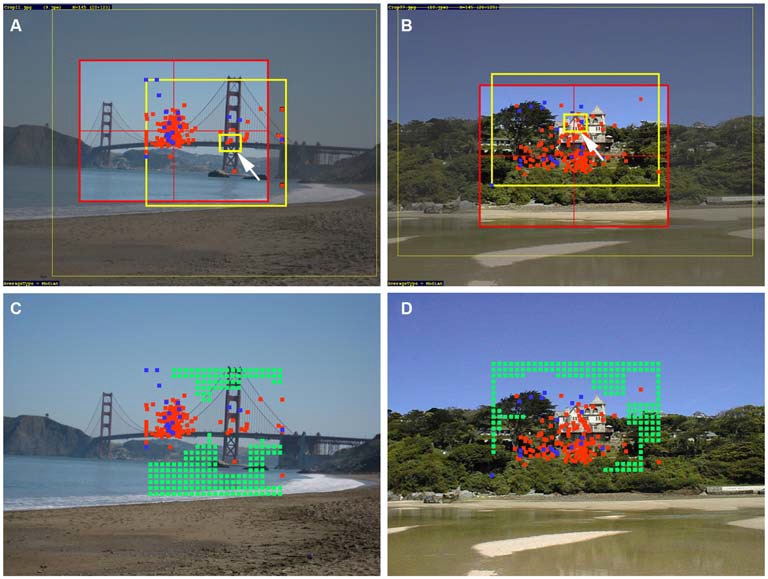
**(A,B)** Examples of the original photographs and experimental results from the study by McManus et al. (2011b). The red rectangles represent the windows that were moved over the original image until participants decided that they had selected a good detail from the image (“cropping window”). Within this window, a fixed area of high interest had to be included, as indicated by the small yellow rectangles (arrows). The red dots represent the center of the details selected by the non-expert participants, the blue dots those selected by the experts. The large yellow rectangles outline the positions of the centers of all possible details in each image. **(C,D)** In addition to the center of details selected by non-experts and experts (red and blue dots, respectively), the center of details that could have been selected but were not (*avoided* details) are indicated by green dots. The photograph shown in **(B,D)** is reproduced with kind permission from Mr. David Grant (© 2015 David Grant).

In the following, we will briefly summarize those parts of the studies by McManus et al. (2011a,b) that relate to the cropping procedure. In brief, non-expert participants (mostly college students) and expert participants (master students of photography at an art college) viewed up to 44 color photographs from various private sources and the internet. The color photographs contained ordinary subject matters, which people would normally photograph, such as family, landscapes and townscapes. They were displayed at their original resolution (1024 × 768 pixels) in landscape format at a viewing distance of about 70 cm (field of view of 24 degrees width) and filled the entire screen. Examples are shown in Figure 1. In an initial viewing phase, participant saw the entire image with a small yellow rectangle (arrows in Figures 1A,B) indicating the focus or subject of the image that had to be included in the subsequent crop. During the cropping phase, participants saw a rectangular part of the image of one quarter of the size of the original image (512 × 384 pixels; large red rectangles in Figures 1A,B), the rest of the screen being black. They could freely move the cropping window around the screen using a computer mouse until they felt that they had achieved a satisfactory crop of the original photograph; this was indicated with a mouse click. A computer program recorded the central positions of the details selected during the cropping. About 71% of the croppings were carried out at first-time presentations and 29% represented repetitions to assess cropping reliability. The same procedure was used for the Mooney versions of a subset of 14 of the original images (see “Introduction” Section; only first-time presentations).

The resulting data formed the basis of our own study, which analyzed a total of 5748 details originally selected by non-experts, and 807 details by experts (Table 1). In order to study whether content played a role in the cropping decisions, we also asked whether the Mooney versions differed with respect to the corresponding saliency maps and eye movement patterns. From the study of McManus et al. (2011b), we analyzed a total of 457 details originally selected by non-experts, and 126 details by experts (Table 1).

**Table 1:** **Deviation from the geometrical image center and Euclidean distance to the image center for all (unmatched) details (in percent of normalized width and height, respectively)**.

		*i*	*n*	Width	Height	Euclidean distance
Color photographs	Non-experts	45	5747	0.096 ± 0.072^1, 2^	0.090 ± 0.064^3^	0.147 ± 0.072^4^
	Experts	35	807	0.114 ± 0.079^1^	0.108 ± 0.088^3^	0.174 ± 0.093^4^
	Avoided	45	5102	0.114 ± 0.069^2^	0.137 ± 0.069^3^	0.193 ± 0.064^4^
Mooney images	Non-experts	14	457	0.120 ± 0.079	0.100 ± 0.070^5,6^	0.170 ± 0.079^7,8^
	Experts	14	126	0.125 ± 0.085	0.124 ± 0.079^5^	0.194 ± 0.084^7^
	Avoided	14	1459	0.111 ± 0.060	0.128 ± 0.070^6^	0.183 ± 0.062^8^

*Values indicate Mean ± SD. i, number of original images; n, number of details. Significantly different from each other (Kruskal-Wallis test with Dunn’s post-test; ^1,2,3,4,6^p < 0.0001; ^5,7,8^p < 0.001)*.

To compare salience and gaze control data between details selected and details that were not selected during cropping (*avoided* details) in the studies by McManus et al. (2011a,b), we mapped all possible cropped details selected by non-experts and experts onto each image. On the original photographs (Figure 1), red dots indicate the geometrical centers of the details selected by non-experts, and blue dots those selected by experts. The large yellow rectangles in Figures 1A,B outline the areas of the central positions of all possible details in each image. To obtain the details that were avoided by non-experts and experts, we discretized the large yellow rectangles in Figures 1A,B by a 64 × 48 grid. As geometrical centers of avoided details, we took all positions that were lying outside the area of squares of 5 × 5 grid points placed on top of the geometrical centers of all selected details in each image. The central positions of the avoided details are indicated by the green dots in Figures 1C,D. A total of 5102 avoided details were analyzed for 45 color photographs and 1459 avoided details for 14 Mooney versions (Table 1). Note that the avoided details were not obtained separately for non-experts and experts because the differences in the number of selected crops between the two groups may have confounded results.

### Eye Tracking

Eye movements were recorded from 34 students, mostly of psychology and medicine (19–38 years old, mean: 23.0, 7 male) who were paid 8 Euros for participating. The Ethics Committee of the University of Jena approved the experiment. Before the experiment, participants were informed about the experimental procedure and agreed to participate by signing a consent form.

The stimuli were 45 original color photographs and 14 Mooney versions that had been selected by both non-experts and experts in the study by McManus et al. (2011b). They were displayed on a BenQ G2200W monitor (resolution 1680 × 1050 pixel) by using the software E-Prime 2.0.822. Before the series of experiments, the monitor was luminance-calibrated (i1Display Pro, X-Rite) and luminance density was set to 200 cd/m^2^. The monitor was positioned at a distance of 74 cm from the eyes in a shaded room with moderate ambient illumination. Presentation of the stimulus was preceded by a fixation cross displayed for 1000 ms. To reduce the influence of the position of the fixation cross on measured eye movements, the crosses were presented in a randomized fashion on either the left, right, top or bottom side of the screen outside the stimulus area. After the fixation cross disappeared, one of the stimuli (1265 × 949 pixel, extending over 25.7 × 20.0 degrees) was shown in a randomized order at the center of the screen for 5000 ms. Then, the next trial was initiated. The participants had no special task and were simply instructed to look at the images. In order to minimize the recognition of objects in the Mooney images, they were presented as the first block, followed by a block with the original color photographs. Each participant viewed every stimulus image once only.

During the presentation of the stimuli, movements of the right eye were recorded using a monocular eye tracker column with chin and forehead rest (SMI iView × HiSpeed 1250). The Person Perception Research Unit, Institute of Psychology, University of Jena, kindly provided the equipment, which uses video-based dark-pupil and corneal reflection tracking (infrared illumination) at a sampling rate of 500 Hz. The eye tracker was 12-point-calibrated for every participant at the beginning of the experiment. If necessary, calibration was repeated to reach an accuracy of <1° average error. The resulting data were prepared for further analysis with the program SMI BeGaze 3.2.28.

### Analysis

**Figure 2:**
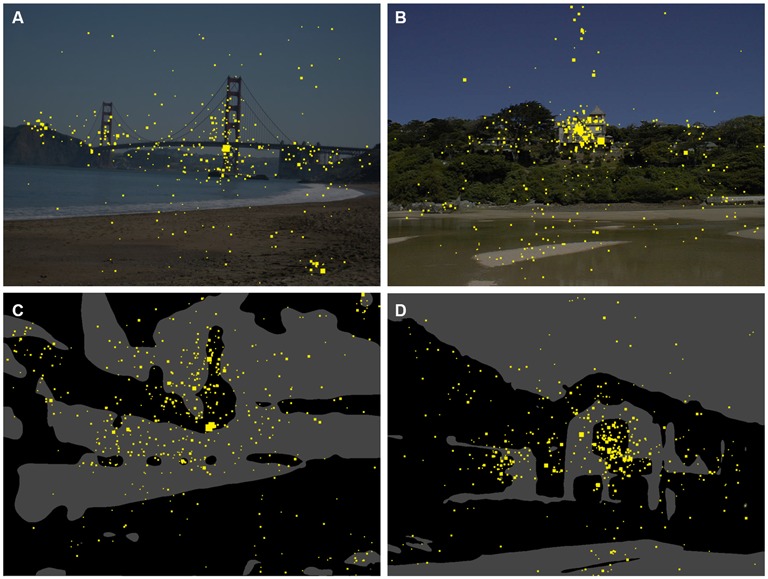
**Gaze maps for the original color images (A,B) and the Mooney images (C,D).** The yellow dots indicate points that participants fixated during the eye tracking study. The size of the dots indicates the total dwelling time for all participants.

To obtain gaze maps, we summed the fixation times for all fixations of an image point for all participants during the entire 5 s viewing periods. The total dwelling times thus obtained were plotted at the respective image co-ordinates onto the images. Figures 2A,B shows examples for the color photographs, and Figures 2C,D for the Mooney images. From these two-dimensional dwelling time plots, we calculated the total dwelling time for regions that corresponded to each of the selected and avoided details in their size and positions.

#### Saliency Maps

Saliency maps were calculated for each of the original images (color photographs and Mooney images) using the ITTI and GBVS methods.

ITTI is based on the use of the Gaussian blur filter in a pyramid and employs center-surround operations to highlight local gradients in 42 intensity, color and orientation feature maps in conjunction with a dynamic neural network (Itti et al., 1998). GBVS exploits graph algorithms and defines Markov chains over various image feature maps, treating the equilibrium distribution over map locations as saliency values (Harel et al., 2007). The two methods were chosen because they are well-established and have previously been applied to a wide variety of problems both in vision research in general and for photographic cropping in particular (Suh et al., 2003; Sun et al., 2009; Wong and Low, 2009; Vaquero et al., 2010; Ardizzone et al., 2013; Borji et al., 2013; Amirshahi et al., 2014; Koide et al., 2015).

From the saliency map of each photograph, we cut out details with positions and size that corresponded exactly to those of the selected and avoided details for this photograph. For each of the details, the sum of saliency was calculated.

#### Matching Details for Euclidean Distance to the Geometrical Image Center

When looking at images displayed on computer monitors, human observers tend to fixate the center of the screen (Bindemann, 2010), regardless of the distribution of the image features (Tatler, 2007). Such a central bias is also incorporated in some of the saliency models (Borji et al., 2013). For example, Harel et al. (2007) consider it as an explicit emergent property of their GBVS method. Saliency maps generated by both the GBVS and ITTI methods show a fall-off of values towards the border of the images. It is therefore likely that, during the cropping procedure, participants tended to select details that are closer to the image center while avoiding details from the periphery of the photographs. Indeed, such a central cropping bias is observed in our data. The details that non-experts and experts selected from color photographs are closer to the geometrical center of the images than the avoided details (Table 1). For Mooney images, a similar difference was found for non-experts but not for expert croppers (Table 1).

Because of the central bias for the cropping decisions, the avoided details, which are from more peripheral regions in the photographs, were expected to have lower total dwelling times and lower saliency values. To counteract this tendency, we matched selected and avoided details with respect to their (Euclidean) distance to the geometrical center. For each photograph, an image was randomly picked from the set of selected details. For this selected detail, an image was randomly drawn from the pool of avoided details until a sample was found that matched its Euclidean distance within a 5% tolerance limit for divergence. The two details were entered into the further analysis as a matched pair and deleted from the set of selected and avoided details, respectively. If no matching avoided detail was found, the selected detail was eliminated from the analysis. The procedure was repeated until all selected details were matched to an avoided detail. The matching procedure was carried out independently for the non-expert details and expert details, respectively. It was also carried out for a pairwise comparison of details selected by non-experts and experts, respectively. The number of images analyzed is listed in Table 2.

**Table 2:** **Deviation from the geometrical image center and Euclidean distance to the image center for pairwise matched details (in percent of normalized width and height, respectively)**.

		*i*	*n*	Width	Height	Euclidean distance
**Color photographs**
Non-expert/avoided	Non-expert	45	1977	0.127 ± 0.073^3^	0.099 ± 0.063^3^	0.178 ± 0.061	
	Avoided	45	1977	0.102 ± 0.064^3^	0.131 ± 0.064^3^	0.180 ± 0.059	
Expert/avoided	Expert	35	517	0.126 ± 0.078	0.112 ± 0.069^2^	0.185 ± 0.072	
	Avoided	35	517	0.116 ± 0.065	0.128 ± 0.070^2^	0.186 ± 0.067	
Non-expert/expert	Non-expert	35	747	0.114 ± 0.080	0.100 ± 0.067	0.161 ± 0.172	
	Expert	35	747	0.112 ± 0.078	0.103 ± 0.068	0.162 ± 0.173
**Mooney images**
Non-expert/avoided	Non-expert	14	336	0.118 ± 0.075^1^	0.097 ± 0.068^1^	0.169 ± 0.071	
	Avoided	14	336	0.102 ± 0.058^1^	0.121 ± 0.070^1^	0.169 ± 0.069	
Expert/avoided	Expert	14	114	0.121 ± 0.085	0.126 ± 0.079	0.193 ± 0.081	
	Avoided	14	114	0.116 ± 0.061	0.135 ± 0.081	0.190 ± 0.075	
Non-expert/expert	Non-expert	14	121	0.136 ± 0.081	0.107 ± 0.078	0.190 ± 0.081	
	Expert	14	121	0.121 ± 0.085	0.120 ± 0.078	0.189 ± 0.082	

*Values indicate Mean ± SD. i, number of original images; n, number of details.^1,2,3^ Significantly different with pairwise comparison (Mann-Whitney test; ^1^p < 0.05, ^2^p < 0.001, ^3^p < 0.0001)*.

#### Statistical Analysis

The mean values for all details that were selected or avoided by non-expert and expert participants, respectively, were calculated for each photograph and Mooney image. Subsequently, mean values were calculated across images. The D’Agostino-Smirnov omnibus normality test was used to assess the normal distribution of the means. If datasets did not pass the normality test, a non-parametric statistical test was used for multiple comparisons (Kruskal-Wallis test with Dunn’s multiple comparison post-test). For paired datasets, a paired *t*-test was carried out for normally distributed data and a Mann-Whitney test for datasets that were not normally distributed. *P*-values smaller than 0.05 were considered significant.

## Results

In this section, we will first address some methodological issues. Second, we will describe differences in total dwelling times and calculated saliency values between details selected during the cropping of photographs (here called *selected* details) and details that were not selected (*avoided* details). Third, we will show that both the dwelling times and saliency values are more balanced for selected than for avoided details. In each case, we will compare the results between the original color photographs and the thresholded (Mooney) versions, and between non-experts and experts.

### Methodological Considerations

To minimize artifacts that are introduced by the central tendency for eye fixations and saliency calculations, we matched the different categories of photographic details (selected by *non-experts*, selected by *experts*, and *avoided* by both) for their Euclidean distance to the geometrical image center in a pairwise fashion (see “Materials and Methods” Section). The resulting matched details did not differ significantly in their Euclidean distance (Mann-Whitney test; Table 2; compare to unmatched original values in Table 1). Differences were, however, observed for the deviations along the *x*-axis and *y*-axis. The mean deviation decreased along the *x*-axis and increased along the *y*-axis.

The same pairwise matching was also carried out for the details selected by non-experts and experts because experts tended to chose details from more peripheral regions of color photographs and Mooney images than non-experts (Table 1). After matching, none of the distances differed significantly (Table 2).

One of the issues in evaluating methods to calculate visual saliency is whether the obtained values can indeed predict eye movements. Here, we address this question by comparing the measures for fixation times and the ITTI and GBVS saliency measures. Specifically, for each detail used in this study, we correlated total dwelling times with saliency values, as well as their respective center-of-mass positions along the *x*-axis and *y*-axis (Table 3). The correlation of total dwelling times with the total saliency values was moderate (range of Spearman coefficients: 0.42–0.48) whereas the correlations between the center-of-mean values for eye movements and saliency were high (range: 0.60–0.86). Generally, high correlations were also observed for the comparison of data obtained by the ITTI and GBVS methods (range: 0.55–0.90; all *p*-values < 0.0001, Table 3).

**Table 3:** **Correlations between eye fixation data and saliency measures (ITTI and GBVS metrics)**.

	Total dwelling time or saliency	CoM-Position (*x* axis)	CoM-Position (*y* axis)
**Color photographs (*n* =11555)**
Eye fixation/ITTI	0.48 (0.46–0.49)	0.797 (0.790–0.804)	0.858 (0.853–0.863)
Eye fixation/GBVS	0.45 (0.43–0.46)	0.622 (0.611–0.634)	0.829 (0.823–0.835)
ITTI/GBVS	0.78 (0.77–0.79)	0.550 (0.537–0.563)	0.768 (0.760–0.776)
**Mooney images (*n* =2042)**
Eye fixation/ITTI	0.42 (0.39–0.46)	0.674 (0.648–0.697)	0.698 (0.674–0.720)
Eye fixation/GBVS	0.42 (0.38–0.46)	0.603 (0.574–0.631)	0.717 (0.695–0.738)
ITTI/GBVS	0.78 (0.77–0.80)	0.871 (0.860–0.881)	0.903 (0.894–0.911)

*The values represent Spearman coefficients r with 95% confidence intervals in parentheses. The correlations for the center-of-mean (CoM) positions are based on their normalized distances to the geometrical image center. All correlations are significant (p < 0.0001). n, number of details*.

**Figure 3:**
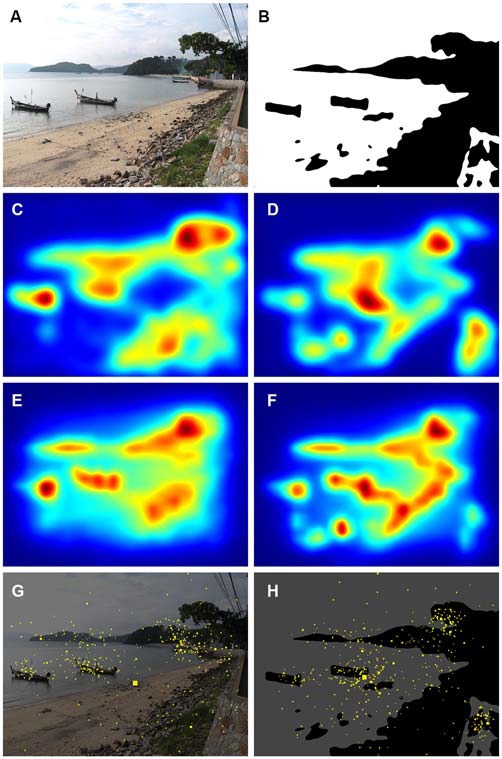
**Comparison of results for an original color photograph (A) and its corresponding Mooney image (B).** The panels below the images show the corresponding saliency maps that were calculated by the ITTI method **(C,D)** and the GBVS method **(E,F)** and the corresponding maps of fixation times **(G,H)**. Saliency values were coded by the rainbow color scale (*blue*, low values; *red*, high values). The yellow dots in the fixation maps indicate points that participants fixated during the eye tracking study; the size of the dots indicates the total dwelling time for all participants. The photograph in **(A)** was downloaded with permission from http://www.knowphuket.com/beaches/CapePanwa.htm.

In Figure 3, the saliency maps (ITTI, Figures 3C,D; GBVS, Figures 3E,F) and the eye fixation data (Figures 3G,H) are compared for an original color photograph (Figure 3A) and the corresponding Mooney image (Figure 3B). Although the saliency maps roughly resemble each other, there are distinct differences for some details in the image. For example, the boat on the left hand side is more prominently represented in all three types of map for the color image. The inverse is true for the wall in the lower right corner of the image. In the present analysis, we therefore calculated values separately for the original color photographs and the Mooney images.

### Total Dwelling Times and Saliency Values are Higher for Selected Details

**Figure 4:**
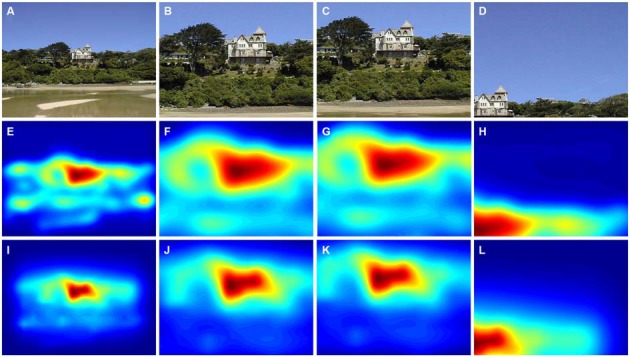
**Results from the cropping of a color photograph and the corresponding saliency maps. (A)** Original photograph. **(B)** A detail selected by a non-expert participant. **(C)** A detail selected by an expert participant. **(D)** A detail avoided by non-experts and experts during the cropping procedure. The panels below the photographs show the corresponding saliency maps that were calculated by the ITTI method **(E–H)** and the GBVS method **(I–L)**. Saliency values were coded by the rainbow color scale (*blue*, low values; *red*, high values). The original photograph in **(A)** is reproduced with kind permission from Mr. David Grant (© 2015 David Grant).

**Figure 5:**
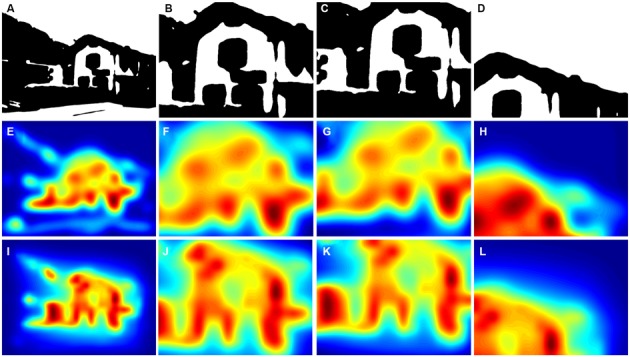
**Results from the cropping of a thresholded binary (Mooney) image and the corresponding saliency maps. (A)** Original image. **(B)** A detail selected by a non-expert participant. **(C)** A detail selected by an expert participant. **(D)** A detail avoided by non-experts and experts during the cropping procedure. The panels below the Mooney images show the corresponding saliency maps that were calculated by the ITTI method **(E–H)** and the GBVS method **(I–L)**. Saliency values were coded by the rainbow color scale (*blue*, low values; *red*, high values).

Figure 2 shows eye fixation maps for representative color photographs (Figures 2A,B) and thresholded binarized (Mooney) images (Figures 2C,D). Figure 4 depicts one of the color photographs (Figure 4A) and the corresponding saliency maps (Figures 4E,I) together with representative details that were selected by a non-expert (Figure 4B) and an expert (Figure 4C), respectively, and an avoided detail (Figure 4D). Figures 4F,G,H,J,K,L show the corresponding details from the saliency map. A similar set of images is shown in Figure 5 for a representative Mooney image.

**Figure 6:**
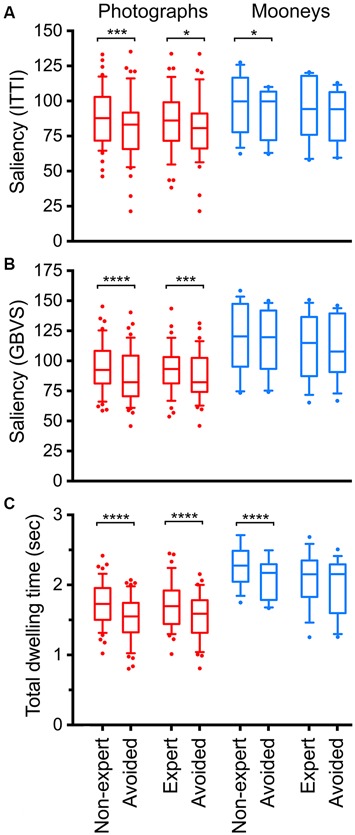
**Calculated total saliency (A,B) and total fixation (dwelling) time (C) in selected and avoided details of original color photographs (*red*) and Mooney images (*blue*).** Saliency was calculated according to the ITTI method **(A)** and the GBVS method **(B)**, respectively. Details were selected by *non-expert* participants, by *expert* participants or they were *avoided* during the cropping procedure, as indicated at the bottom of panel **(C)**. The boxes comprise the percentiles between 25% and 75% with the median value indicated by the horizontal line within each box. The whiskers represent the 10 and 90 percentiles. Significant differences between the selected and avoided details are indicated by the asterisks (*p < 0.05; ***p < 0.001; ****p < 0.0001).

Figure 6 summarizes the results for all photographs (red) and all Mooney images (blue). Figure 6A shows results for the ITTI method, Figure 6B for the GBVS method, and Figure 6C for the total dwelling time.

First, we compared results for the original color photographs with the Mooney images. The total dwelling times were systematically higher for the Mooney images compared to the photographs, both for details selected by non-experts and experts participants, as well as for the avoided details (Figure 6; two-tailed *t*-test, *p* < 0.0025). No such differences were obtained for the ITTI saliency values (*p*-values between 0.08 and 0.17). GBVS saliency values were lower for the photographs (*Mean*: 93.3 ± 18.8 *SD*) than for the Mooney images (*Mean*: 113.1 ± 28.0 *SD*; two-tailed *t*-test, *df*: 13, *p* = 0.032), but only for the details selected by experts. The GBVS values of the details avoided by both non-experts and experts were also higher for the Mooney images than for the photographs (*df*: 13; *p* = 0.034 and *p* < 0.019, respectively).

Second, the selected and avoided details from the original color photographs were compared. For the color photographs, saliency values calculated according to the ITTI method (Figure 6A) were significantly higher for details selected by non-experts (*Mean*: 88.4 ± 20.4 *SD*) than for the avoided details (*Mean*: 80.4 ± 23.0 *SD*; two-tailed *t*-test, *df*: 44, *p* = 0.0006). The same holds true for details selected by experts (*Mean*: 86.8 ± 22.5 *SD*; compared to *Mean*: 80.2 ± 23.2 *SD* for avoided details; *df*: 34, *p* = 0.015).

Saliency calculated according to the GBVS method (Figure 6B) yielded similar differences for the comparison of details selected and avoided by non-experts (*Mean*: 95.6 ± 20.9 *SD* for selected details *vs. Mean*: 86.9 ± 21.6 *SD* for avoided details; *df*: 44, *p* < 0.0001) and details selected and avoided by experts (*Mean*: 93.3 ± 18.8 *SD* for selected details *vs. Mean*: 86.6 ± 19.4 *SD* for avoided details; *df*: 34, *p* = 0.0002).

Results for the total dwelling time (Figure 6C) were similar to those obtained by calculating saliency. Dwelling times were higher for details selected by non-experts (*Mean*: 1.71 ± 0.32 *SD*) than for avoided details (*Mean* 1.52 ± 0.32 *SD*; *df*: 44, *p* < 0.0001), and details selected by experts (*Mean* 1.70 ± 0.34 *SD*) than for avoided details (*Mean* 1.52 ± 0.32 *SD*; *df*: 34, *p* < 0.0001).

Next, we compared values for the selected and avoided details from the Mooney images. The only significant difference was that Mooney images showed higher ITTI values for the details selected by non-experts (*Mean*: 99.3 ± 21.4 *SD*) than the avoided details (*Mean*: 91.9 ± 18.0 *SD*; *df*: 13, *p* = 0.011), and higher dwelling times for the details selected by non-experts (*Mean* 2.27 ± 0.29 *SD*) than the avoided details (*Mean* 2.09 ± 0.29 *SD*; *df*: 12, *p* < 0.0001).

Finally, we compared the values for non-expert and expert participants. Because the details selected by experts were from more peripheral regions than those selected by non-experts on average (Table 1; see “Materials and Methods” Section), we also analyzed details that were matched pairwise for their Euclidean distance to minimize the effect of central bias (see above). GBVS saliency values were higher for details selected by non-experts (*Mean* 96.8 ± 18.9 *SD*) than by experts (*Mean* 95.7 ± 18.8 *SD*; *df*: 34, *p* = 0.024; Figure 5). No other comparisons yielded significant differences.

In summary, participants tended to select details that contained higher visual saliency and were fixated longer than details that were avoided during cropping.

### Dwelling Time and Saliency Show a More Balanced Distribution in Details Selected During Cropping

As a measure of visual balance, we chose to analyse the distribution of dwelling (fixation) times on each detail because, as a behavioral measure, it may relate to the subjective feeling of visual balance in images more closely than simpler physical measures, such as the distribution of luminance, which has been analyzed previously (McManus et al., 2011b). Our intuition was that a well-balanced image would attract eye movements equally strongly to its left and right halves as well as to its upper and lower halves. In parallel, the distribution of saliency was calculated by the ITTI and the GBVS methods to assess how well the calculated saliency measures can predict the behavioral data. To quantify the balance, the center-of-mean of the distribution of these measures was determined for each detail. Consequently, the measures were distributed in a well-balanced fashion if the center-of-mean was close to the geometrical center of the details. The more the center-of-mean deviated from the geometrical image center, the more the distribution was out of balance.

**Figure 7:**
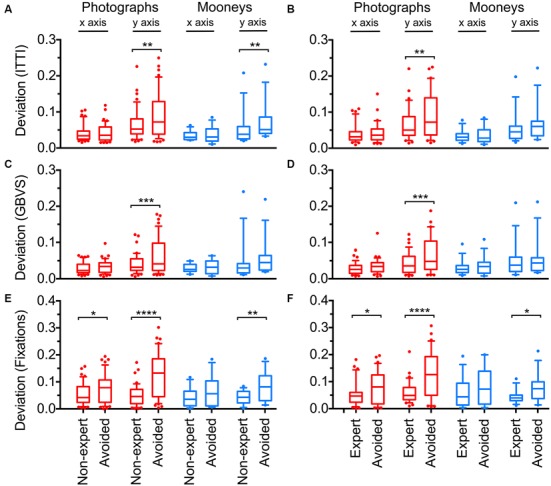
**Deviations of the center-of-mean values from the geometrical image centers for the distribution of calculated saliency (A–D) and fixation (dwelling) time (E,F).** Saliency was calculated according to the ITTI method **(A,B)** and the GBVS method **(C,D)** respectively. Details were selected by *non-expert* participants, by *expert* participants or they were *avoided* during the cropping procedure, as indicated at the bottom of panels **(E,F)**. *Red* indicates results for original color photographs and *blue* for Mooney images. Results are shown separately for the *x-axis* and the *y-axis*. The boxes comprise the percentiles between 25% and 75% with the median value indicated by the horizontal line within each box. The whiskers represent the 10 and 90 percentiles. Significant differences between the selected and avoided details are indicated by the asterisks (*p < 0.05; **p < 0.01; ***p < 0.001; ****p < 0.0001).

Figure 7 shows the average deviations of the center-of-means for the distributions of the ITTI saliency measure (Figures 7A,B), the GBVS measure (Figures 7C,D) and the total dwelling times (Figures 7E,F). Data for non-experts are shown on the left-hand side (Figures 7A,C,E) and for experts on the right-hand side (Figures 7B,D,F).

In general, the three measures showed similar deviations for non-experts and experts. For the color photographs, deviations were smaller for the selected details than for the avoided details along the *y*-axis. The deviation for ITTI (Figures 7A,B) was smaller for the details selected by non-experts (*Mean*: 0.066 ± 0.042 *SD*) than for avoided details (*Mean* 0.088 ± 0.063 *SD*; *df*: 44, *p* = 0.0027). The same was found for details selected by experts (*Mean* 0.067 ± 0.045 *SD*) when compared to avoided details (*Mean* 0.089 ± 0.063 *SD*; *df*: 34, *p* = 0.0092). No significant difference was found for deviations along the *x*-axis.

Similar results were obtained for GBVS (Figures 7C,D). Again, non-experts selected details that deviated less from the geometrical image center (*Mean*: 0.041 ± 0.026 *SD*) than the avoided details (*Mean* 0.062 ± 0.048 *SD*; *df*: 44, *p* = 0.0006), as did the experts (*Mean*: 0.044 ± 0.029 *SD* vs. *Mean*: 0.065 ± 0.048 *SD*; *df*: 34, *p* = 0.0004).

Deviations for eye fixations (Figures 7E,F) were different not only along the *y*-axis, but also along the *x*-axis. For non-experts, deviations were smaller for the selected details than for avoided details along the *y*-axis (*Mean*: 0.050 ± 0.039 *SD* for selected details *vs. Mean*: 0.123 ± 0.083 *SD* for avoided details; *df*: 44, *p* < 0.0001) and along the *x*-axis (*Mean*: 0.054 ± 0.041 *SD* vs. *Mean*: 0.077 ± 0.054 *SD*; *df*: 44, *p* = 0.032). For experts, results were similar (for *y*-axis: *Mean*: 0.060 ± 0.040 *SD* vs. *Mean*: 0.125 ± 0.088 *SD*; *df*: 34, *p* < 0.0001; and for *x*-axis: *Mean*: 0.054 ± 0.047 *SD* vs. *Mean*: 0.078 ± 0.058 *SD*; *df*: 34, *p* = 0.036).

**Figure 8:**
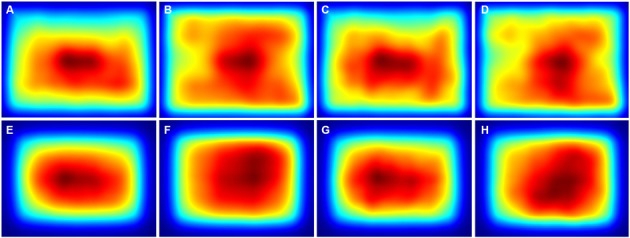
**Averaged saliency maps for all details from the color photographs that were selected by non-expert participants (A,E) and expert participants (C,G) and for the respective avoided details after pairwise matching with the non-expert details (B,F) and the expert details (D,H), respectively.** The maps were calculated with the ITTI method **(A–D)** and the GBVS method **(E–H)**, respectively. Saliency values were coded by the rainbow color scale (*blue*, low values; *red*, high values).

The averaged saliency maps for the color photographs (Figure 8) confirmed these observations. Compared to the details selected by non-experts (Figures 8A,E) and experts (Figures 8C,G), high saliency values were distributed more widely along the *y*-axis for the avoided details (Figures 8B,F,D,H).

**Figure 9:**
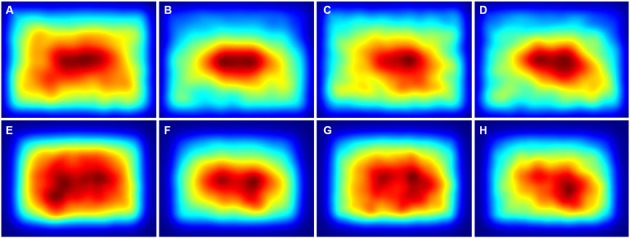
**Averaged saliency maps for all details from the thresholded binary (Mooney) images that were selected by non-expert participants (A,E) and expert participants (C,G) and for the respective avoided details after pairwise matching with the non-expert details (B,F) and the expert details (D,H), respectively**. The maps were calculated with the ITTI method **(A–D)** and the GBVS method **(E–H)**, respectively. Saliency values were coded by the rainbow color scale (*blue*, low values; *red*, high values).

For the Mooney images, fewer differences were observed, and only for the *y*-axis. Deviations for the ITTI method (Figures 7A,B) were smaller for details selected by non-experts (*Mean*: 0.054 ± 0.049 *SD*) than for the avoided details (*Mean* 0.073 ± 0.053 *SD*; *df*: 12, *p* = 0.0086). Deviations for fixation times (Figures 7E,F) were smaller for details selected by both non-experts and experts (for non-experts: *Mean*: 0.043 ± 0.025 *SD* vs. *Mean*: 0.085 ± 0.055 *SD*; *df*: 12, *p* = 0.0075; and for experts: *Mean*: 0.045 ± 0.025 *SD* vs. *Mean*: 0.079 ± 0.053 *SD*; *df*: 13, *p* = 0.043). In the averaged saliency maps for the Mooney images (Figure 9), differences were less prominent than for the color photographs (Figure 8).

The comparison between data from non-experts and experts did not result in significant differences after pairwise matching for Euclidean distance (data not shown).

In summary, for both calculated visual saliency and eye fixation times, the selected details tended to be more balanced than the avoided details, in particular along the *y*-axis.

## Discussion

In the present study, we identified two perceptual features that played a role when participants cropped a photograph. First, participants generally selected regions during cropping that displayed a relatively high degree of visual saliency and longer dwelling times. This result was expected because salient regions are, by definition, those that attract visual attention and therefore, participants tend to include them in the selected detail in most cases (Ardizzone et al., 2013). Second, calculated visual salience and dwelling times are distributed in a well-balanced manner in the selected details. This result relates to previous studies on pictorial balance. Following the discussion of this relation in the next section, we will review the factors that influence pictorial balance and how our results can be used in automated cropping methods.

### Pictorial Balance, Saliency and Gaze Behavior

In the present work, we analyzed the balance of visual saliency, a relatively complex measure that combines several low-level visual features (Itti et al., 1998; Harel et al., 2007). Reanalysing the image material from the studies by McManus et al. (2011a,b), we demonstrate that participants selected photographs during cropping so that the center-of-mean for saliency was close to the geometrical image center (Figure 7). This finding is mirrored by the behavioral data. For the dwelling times, the center-of-means was even more consistently centered around the geometrical image center than calculated saliency. A good correlation between eye movement and saliency data was also found for total saliency and total dwelling times and for the y- and x-positions of the center-of-means (Table 3). These results underline the general usefulness of saliency measures in predicting specific aspects of gaze behavior (Borji et al., 2013).

According to Locher et al. (1996), pictorial balance is achieved when the elements of a painting are positioned about a balance center so that the elements seem to be anchored and stable. Such balance judgments are interpreted to be the result of a global integration of information across the entire picture field (McManus et al., 1985), especially from its central region (Locher et al., 1996). Moreover, there is evidence that the center of subjective balance tends to be closely aligned with the geometric center (midpoint) of the image (Locher et al., 1998), regardless of element type, format, or phase of construction of a visual display created by participants (Locher et al., 2001). This applies also for color, as shown for paintings by Mondrian (Locher et al., 2005). Our present findings are compatible with this notion and suggest saliency as an objective measure that contributes to pictorial balance. Whether this measure is useful also for other types of images (for example, artworks) remains to be studied.

### Specific Image Content and Perceptual Factors Affect Pictorial Balance

Two different types of information may influence the subjective assessment of visual balance: Specific content displayed in the images, and the visual structure of the images, i.e., low- or mid-level perceptual features.

The dependance of balance on perceptual features has been assessed in studies that compare experts on art or photography and naïve (non-expert) participants. For example, Locher (2003) suggested that the visually right structure of artworks (i.e., the “good” composition) can be recognized not only by art experts, but also by viewers who lack formal training in the visual arts. Accordingly, pictorial balance assessments by art or design experts are in good agreement with those of non-expert viewers (Locher et al., 1996, 1999). Supposedly, non-expert participants are less prone to take into account specific image content in their judgments and as a consequence, they may rely more on formal compositinal properties (Koide et al., 2015). This notion is supported by results from the present study where the cropping decisions of non-experts were more clearly related to the saliency and eye movement measures than those of the experts (Figures 6, 7). This result is also consistent with the observation by McManus et al. (2011b) that the crops of experts were from positions that scattered more widely across the original photographs. The same study also carried out a qualitative analysis of the reasons participants gave for their cropping decisions. Non-expert participants more often mentioned image content, whereas experts mentioned formal compositional properties. Balance in artworks can also be recognized rapidly and effortlessly (McManus et al., 1985), even “at a glance” with exposure times of as short as 100 ms duration (Locher and Nagy, 1996), which may be too fast for extensive top-down processing based on semantic information. Together, these and previous findings (Koide et al., 2015) indicate that experts base their cropping decisions on semantic information to a larger extent than non-experts. Alternatively, experts may use different perceptual features that are not appreciated by non-experts. This explanation, however, seems less likely because the general results for saliency and eye movements were similar for experts and non-experts.

Of interest is our finding that the differences between selected and avoided details are more pronounced along the *y*-axis than along the *x*-axis (Figure 7). In other words, in the study by McManus et al. (2011b), moving the cropping window to the left side or to the right side changed visual balance less than moving the cropping window up and down. Possibly, this is because the horizon extends horizontally (for example, see photographs in Figure 1), so that the visual structure changes less horizontally than vertically in photographs of natural scenes. Alternatively, there may be intrinsic perceptual differences between the left/right and upper/lower halves of our perceptual visual field. For example, confirming earlier studies, Niekamp (1981) found that the upper half of a visual field has inherently greater visual weight than the lower half of stimuli that consisted of simple geometrical forms; he did not obtain similar differences between the left and right sides of the images. McManus et al. (1985) showed that objects in the upper part of an image have greater weight than those in the lower part, and objects on the right part have greater weight than on the left part.

The effect of content and perceptual features can be assessed also by comparing the original photographs and their corresponding Mooney images. Because participants viewed the Mooney images before the color photographs, it was hard for them to recognize content in the Mooney images, which represented rather abstract patterns to them. We did observe differences for saliency and gaze behavior (Figure 3), but the general pattern of cropping decisions was similar: For both types of image, participants preferred details that included highly salient regions in a balanced fashion. This result indicates that participants used similar criteria for cropping images with content and largely devoid of content. A detailed analysis of the differences between original and Mooney versions of the photographs is beyond the scope of the present study.

Besides perceptual features, semantic information can affect gaze behavior and visual behavior (see “Introduction” Section). For example, in the case of paintings, information about the title of a painting can have an influence both on eye movements and on the distribution of fixation times over different regions of the painting (Kapoula et al., 2009). Balance on its own does not suffice to distinguish between masterworks of art and paintings of lesser artistic quality (Vartanian et al., 2005). Moreover, for the field of architecture, Hasse and Weber (2012) reported that visual balance does not affect beauty judgments of facades. For artworks, it has been suggested that both perceptual and cognitive processing contribute to aesthetic experience (Redies, 2015).

### Relevance of Results for Automated Cropping Methods

Our study provides experimental support for the notion widely held in computer vision that saliency calculations can be employed in automated cropping procedures to improve the aesthetic outcome (Santella et al., 2006; Liu et al., 2010; Jahanian et al., 2015; Wang et al., 2015). In a well-controlled psychological experiment, we demonstrate that the centering of saliency mass onto the geometric image center results in images that are preferred by viewers compared to more unbalanced images from the same photograph. This principle has already been used in some cropping procedures as *a priori* knowledge. For example, Liu et al. (2010) used saliency maps and applied several principles of image composition, including pictorial balance, to automatically select photographic details that were aesthetically more pleasing.

The usefulness of saliency calculations, however, depends on the metric chosen for a given application. While the two methods used in the present study (ITTI, Itti et al., 1998; GBVS, Harel et al., 2007) did not differ much in their predictive power, we obtained less satisfactory results with a third method (frequency-tuned salient region detection; Achanta et al., 2009; data not shown). Despite the general usefulness of saliency calculations in automated cropping procedures, viewers still prefer crops that are based on actual gaze behavior (Santella et al., 2006), possibly because actual eye movement data more closely relate to subjective preferences than calculated predictions of gaze behavior.

## Author Contributions

JA, LF, HK and CR conceived the experiments; ICM provided the dataset of photographs and unpublished experimental data; SAA, MK and CR developed the computer programs for the analysis; JA and LF carried out the experiments; JA, LF and CR analyzed the data; CR wrote the first version of the manuscript; and SAA, MK, ICM, HK and CR contributed to the final version of the manuscript.

## Conflict of Interest Statement

The authors declare that the research was conducted in the absence of any commercial or financial relationships that could be construed as a potential conflict of interest.
